# Conclusive Thoughts for a New Beginning

**DOI:** 10.3390/children10010060

**Published:** 2022-12-27

**Authors:** Matteo Chiappedi

**Affiliations:** Vigevano Child Neurology and Psychiatry Unit, ASST Pavia, 27100 Pavia, Italy; matteo_chiappedi@asst-pavia.it

When I was asked to name this Special Issue, I was both honored and worried, as being appointed the Guest Editor was a significant achievement and honor. Although I had been trained as a child neuropsychiatrist (as in Italy, pediatric psychiatry and neurology are still joined in one discipline), my main interest has always been child psychiatry. Thus, I was worried that relatively few papers concerning child and adolescent psychiatry had previously been published in *Children*. However, many things have changed since this Special Issue was created under the Child Neurology Section (which was, at that time, the best fit available) and now, a Child and Adolescent Psychiatry Section exists as a part of *Children*.

Since the previous Editorial concerning this Special Issue [1], many interesting manuscripts have been submitted. This was surprising, since a number of other Special Issue had been opened and some of them potentially overlap with this wide topic I had chosen (“Recent Advances in Child and Adolescent Psychiatry”). When the Special Issue was closed, 33 manuscripts had been submitted and 20 had been accepted (this does not include the above mentioned Editorial [1]). As the Guest Editor, I am privileged to have the opportunity to read these interesting contributions and to choose those that I (and the wonderful colleagues that provided their service as reviewers) believed were not only scientifically sound (which was true for all submitted manuscripts), but also of a high interest for the wide readership of *Children*.

Dr. Bonete and coworkers provided data concerning the development of the Interpersonal Problem-Solving Skills Program [2]. They discussed the use of a task meant to increase the generalization of interpersonal problem-solving skills in adolescents and young adults with autism spectrum disorder who showed a reduced ability to imagine different possible scenarios. Their findings showed that this improved after treatment.

Dr. Chen and coworkers studied the psychometric properties of the Child and Adolescent Mindfulness Measure (CAMM) in Chinese primary school students [3]. Mindfulness has become a trending topic in psychiatry over the last 10 years, and this study combined a description of its role in physical and mental health with a rigorous psychometric analysis. Although conducted in a rather specific population, this was a good example of what is expected to happen before a tool is used in psychiatry (and in medicine in general).

The interaction between a physical disease, psychological factors and health-related quality of life (HRQoL) was the focus of the paper by Dr. Liu and coworkers [4]. They studied children with congenital heart disease to show that there was a higher impact on patients’ psychological adjustment and HRQoL in those with cyanotic heart disease, although the need for invasive treatments was another relevant factor.

The Mondino Foundation Eating Disorders Clinical and Research Group contributed a manuscript concerning the interactive patterns in families of patients with restrictive eating disorders [5]. They used the clinical version of the Lausanne Triadic Play to assess family functioning and confirmed the presence of a collusive alliance in these families, but expanded the existing literature on this topic by showing a correlation between this interactive pattern and a higher global score for alexithymia in the patient, according to the Toronto Alexithymia Scale.

The paper written by Dr. Vigil-Dopico and coworkers [6] deals with a highly relevant topic for child psychiatry, i.e., play. The authors studied a significant number of children to assess play performance and compared their performance with internalizing and externalizing problems that were reported by their parents through the Strengths and Difficulties Questionnaire. A relationship was found between reduced play performance and higher psychosocial problems. The authors provide a tentative explanation, assuming that executive functions could have a role in self-regulation (in particular, during play performance) and in social cognition.

A cross-sectional study conducted in some of the most important pediatric departments in Italy during the so-called “first lockdown” (i.e., the first application of the stay-at-home order) was presented by Dr. Correale and coworkers [7]. They evaluated pediatric patients with different forms of chronic illness hospitalized from March to May 2021 and found a high proportion of significant depressive and anxious symptoms (reported by roughly two out of three children). This highlighted the need to study the effect of the COVID-19 pandemic (and of the related preventive measures) in the most vulnerable subjects.

A more positive view of the effects of the first phase of the COVID-19 pandemic on adolescents was provided by Malerba and coworkers [8]. In their longitudinal study on a sample of Italian adolescents, they found, on average, a good adjustment, which seemed to be mainly associated with a high degree of positivity as a trait, a factor that could have somehow overweighted the difficulties caused by the many uncertainties that emerged in those months.

It is rather evident that the COVID-19 pandemic had a significant influence on this Special Issue, not only because it was started in 2020, but also because 4 research papers [7,8,9,10] out of the 20 published explored aspects connected with it. Since the epidemiologic situation seems to be evolving favorably, although some concerns are still present, one could begin to conduct research on the factors that acted as facilitators or as barriers, on the new models of intervention (exploiting technology to vehiculate treatments and support) and on the psychic scars left from these years. However, the fact that 16 research papers, even in such unprecedented times, addressed relevant aspects of child and adolescent psychiatry independent from the COVID-19 pandemic shows that there is a lot more to yet explore. Maybe, in a relatively short time, a new Special Issue will be needed.
